# Prediction of Hearing Help Seeking to Design a Recommendation Module of an mHealth Hearing App: Intensive Longitudinal Study of Feature Importance Assessment

**DOI:** 10.2196/52310

**Published:** 2024-08-12

**Authors:** Giulia Angonese, Mareike Buhl, Inka Kuhlmann, Birger Kollmeier, Andrea Hildebrandt

**Affiliations:** 1 Cluster of Excellence Hearing4All Oldenburg Germany; 2 Psychological Methods and Statistics Lab Department of Psychology Carl von Ossietzky Universität Oldenburg Oldenburg Germany; 3 IHU reConnect, Institut de l’Audition, Fondation Pour l'Audition, Inserm, AP-HP Institut Pasteur Université Paris Cité F-75012 Paris France; 4 Department of Medical Physics and Acoustics Carl von Ossietzky Universität Oldenburg Oldenburg Germany; 5 Research Center Neurosensory Science Carl von Ossietzky Universität Oldenburg Oldenburg Germany

**Keywords:** hearing loss, mobile health, mHealth, older adults, help seeking, mobile study, machine learning, supervised classification, feature importance, profiling, mobile phone

## Abstract

**Background:**

Mobile health (mHealth) solutions can improve the quality, accessibility, and equity of health services, fostering early rehabilitation. For individuals with hearing loss, mHealth apps might be designed to support the decision-making processes in auditory diagnostics and provide treatment recommendations to the user (eg, hearing aid need). For some individuals, such an mHealth app might be the first contact with a hearing diagnostic service and should motivate users with hearing loss to seek professional help in a targeted manner. However, personalizing treatment recommendations is only possible by knowing the individual’s profile regarding the outcome of interest.

**Objective:**

This study aims to characterize individuals who are more or less prone to seeking professional help after the repeated use of an app-based hearing test. The goal was to derive relevant hearing-related traits and personality characteristics for personalized treatment recommendations for users of mHealth hearing solutions.

**Methods:**

In total, 185 (n=106, 57.3% female) nonaided older individuals (mean age 63.8, SD 6.6 y) with subjective hearing loss participated in a mobile study. We collected cross-sectional and longitudinal data on a comprehensive set of 83 hearing-related and psychological measures among those previously found to predict hearing help seeking. Readiness to seek help was assessed as the outcome variable at study end and after 2 months. Participants were classified into help seekers and nonseekers using several supervised machine learning algorithms (random forest, naïve Bayes, and support vector machine). The most relevant features for prediction were identified using feature importance analysis.

**Results:**

The algorithms correctly predicted action to seek help at study end in 65.9% (122/185) to 70.3% (130/185) of cases, reaching 74.8% (98/131) classification accuracy at follow-up. Among the most important features for classification beyond hearing performance were the perceived consequences of hearing loss in daily life, attitude toward hearing aids, motivation to seek help, physical health, sensory sensitivity personality trait, neuroticism, and income.

**Conclusions:**

This study contributes to the identification of individual characteristics that predict help seeking in older individuals with self-reported hearing loss. Suggestions are made for their implementation in an individual-profiling algorithm and for deriving targeted recommendations in mHealth hearing apps.

## Introduction

### Mobile Health Solutions for Hearing Care

Hearing enables individuals to experience their surroundings and communicate with others. Thus, hearing difficulties can have a strong impact on individuals’ quality of life. Hearing loss (HL) is one of the most common chronic diseases worldwide and affects 20.3% of the world’s population. More than 60% of individuals with HL are aged >50 years, with the principal cause being age-related HL [1]. Untreated hearing difficulties have been associated with lower self-rated health, depression, and anxiety in addition to physical and cognitive decline, dementia, and hospitalization in the older population [2-4]. The primary rehabilitative strategy for individuals with moderate to severe HL is the use of hearing aids (HAs), which increase activity levels, general health, and quality of life [5] and decrease social isolation and depressive symptoms [6] by supporting hearing ability and communication efficacy. Despite the positive effects of hearing rehabilitation, the prevalence of HA use is still limited to approximately 25% of the population with hearing impairment [2,4,7,8]. Moreover, there is an average delay of 9 years between the time a person first acknowledges hearing difficulties and the first contact with a hearing health professional [9].

Developing easily accessible and affordable mobile health (mHealth) solutions in audiology would promote broader and faster access to diagnosis and health services, fostering an early rehabilitation and reducing the impact of HL on the individual. It is estimated that 55% of the global population and 87% of the European population will use internet services on a mobile device by 2030 [10]. Thus, it is evident that mHealth solutions have the potential to significantly impact behaviors in the population. Current studies indicate that the use of tablets and smartphones, including mHealth apps, is steadily increasing among adults aged ≥65 years [11-13], who are even reported to be the fastest-growing population of smartphone users [11]. In the last decades, various mHealth apps have been developed for ear and hearing assessment [14-18] and for HA rehabilitation [19,20], in addition to tele-audiology services [21] and hybrid clinics [22]. The ease of use and accessibility of mHealth solutions can have an impact on self-awareness and recognition of HL and foster knowledge and use of in-person services [19,23,24]. Moreover, mHealth solutions show the potential to promote more equitable health care in low- and middle-income countries where access to health care facilities and professionals is limited [16,25]. Finally, mHealth apps that are quick and easy to use in everyday life have the potential to provide clinicians with important information at both the diagnostic and intervention phases and can help explore and understand daily experiences of the user with HL and facilitate more timely responses [19].

An mHealth hearing app might become the first contact with a hearing diagnostic service and, therefore, should motivate individuals in need to seek professional help in a personalized manner to maximize the impact of early health services on the population. However, even though professional support might be recommended given the hearing test result, many users might still be hesitant to seek help. This is particularly true among the older adult population, in which HL can be slow and gradual and is often considered a natural aspect of aging. In addition, individuals may not be aware of the rehabilitation options and hearing health care services available to them or how to access them [4]. Some users may even choose to ignore indications to seek help for various reasons, such as low awareness of HL or stigma associated with HA use. In addition, individuals high on neuroticism and low on agreeableness might generally distrust external advice. Acquiring more information about users’ personal characteristics and generating their individual profiles can inform the creation of targeted recommendations, particularly for those users who need more convincing incentives to take action. These recommendations can aid skeptical individuals in their decision-making process. For example, repeated feedback on daily hearing tests could increase self-awareness of HL, and HA simulators could promote positive expectations regarding HAs. Moreover, information about users’ personal characteristics collected via an mHealth hearing app could later assist clinicians in providing personalized counseling. It follows that the assessment of a person with hearing difficulties needs to go beyond the simple quantification of HL and should also take into account individual characteristics that have been shown to influence the readiness of individuals to seek professional help [4,26].

### Predictors of Hearing Help Seeking and HA Uptake

Hearing help seeking can be seen as a first crucial step toward the decision to take up an HA [26]. Help seeking takes place after a contemplation stage [3] where a listener in need is initially ambivalent about making changes. Help seekers would then prepare (seek information and plan) and take action [3] toward a change in behavior and attitude, namely, consulting a health care professional about their hearing difficulties [4,27]. Acknowledgment and acceptance of hearing difficulties and their impact on everyday life have been discussed as the most important predictive features of hearing help seeking as well as later HA uptake [5,6,26,28,29]. In the older adult population, HL is frequently perceived as part of the natural aging process, and other health issues are prioritized for treatment [4,30]. Even when HL is identified, individuals might reject the use of an HA due to expected costs, stigma, and negative stereotypes [5,7,28]. However, a positive attitude toward HAs [26,27], high expectations to benefit from them [26,29], and perceived self-efficacy in their daily management [17,18] were shown to promote help seeking and HA uptake. Other relevant covariates that have been identified in the literature are personal attitudes, beliefs, and personality traits. Individuals who are more prone to seeking help and successfully uptake an HA show higher internal locus of control [2,17], self-efficacy [31,32], and agreeableness as well as lower neuroticism and openness [2]. Altogether, such individual characteristics refer to a general self-confidence in the ability to cope with critical situations, good acceptance of others’ suggestions and recommendations, and less susceptibility to shame and embarrassment.

Given the wide range of traits and behaviors that have been reported in the literature to be associated with hearing help seeking and HA uptake, predictive models can be developed that take into account the multifaceted nature and association patterns of these traits and behaviors. Machine learning models are built on a large number of predictors simultaneously, usually leading to more accurate predictions than univariate or smaller models that take into account only a limited number of predictors. These models can capture complex and nonlinear relationships between the outcome and its predictors. In addition, when combined with cross-validation (CV) approaches, they draw more robust conclusions and generalize to new data. Currently, the use of machine learning to support health care applications is rapidly growing, particularly in the areas of predictive analytics, diagnosis and treatment, personalized medicine, clinical decision support, and population health management [33]. Machine learning algorithms and feature importance analysis can be used to identify the most relevant predictors of hearing help seeking from the many features hitherto reported in the literature. In an mHealth context, it is crucial to limit the number of features to a small set of key predictors to create a concise and efficient assessment battery.

### Rationale and Objectives

This study aimed to identify the most important predictive features for hearing help seeking, planning to design an individual-profiling module for an mHealth hearing app. Such a module will categorize help seekers versus nonseekers and, ultimately, inform the design of targeted or even personalized treatment recommendations. For this purpose, data from a large number of questionnaires and tests covering different hearing-related and psychological characteristics, together with multiple assessments of a hearing screening test, were collected in a longitudinal study that simulated an mHealth hearing app. To target potential users of future apps of this kind, this study was geared toward individuals with subjectively perceived hearing difficulties who had not yet been compensated with HAs. We selected 25 assessment tools based on an extensive literature review of covariates of hearing help seeking and HA uptake. From these, a comprehensive set of 83 features was derived. We used supervised machine learning algorithms to predict the readiness to seek professional help as assessed at the end of the study and after 2 months as self-reports on intention to seek help. Feature importance analysis was used to narrow down the large number of features and identify a small set of key traits to predict hearing help-seeking behavior. On the basis of these results, we aimed to derive suggestions for the implementation of a profiling module as a short and concise assessment battery that can be administered after an audiological test. This could be included in existing or future mHealth apps in a modular manner. Knowledge of a user’s propensity to seek help can be used to provide specific recommendations to encourage the use of hearing health care services. Ultimately, our aim was to provide clinicians and mHealth app developers with relevant knowledge about personal characteristics that are helpful in promoting hearing health by encouraging the uptake of hearing health care services and HAs when needed.

The following research questions (RQs) guided our study design and analyses:

1. Which machine learning model can best predict help seeking and categorize individuals into help seekers versus nonseekers? (RQ 1)
2. Which hearing-related and psychological features are most relevant to classify individuals into help seekers versus nonseekers? (RQ 2)
3. How can feature importance measures inform the design of targeted recommendations for users of a future mHealth hearing app? (RQ 3)

## Methods

### Participants

Adults aged >50 years were recruited between August 2021 and August 2022 through the Ebay minijob announcement web platform and the university intranet and via mailing list services of several German universities’ guest audience and senior programs. The inclusion criteria were subjective reports of hearing difficulties in daily life, ownership of and ability to use a smartphone, and good command of the German language. The exclusion criterion was the use of HAs. A total of 192 individuals were enrolled in the study. In total, 3.6% (7/192) of the participants dropped out during the study, resulting in a completion rate of 96.4% (185/192). The final data set included 185 participants—106 (57.3%) female and 79 (42.7%) male (0 diverse)—aged between 47 and 82 years, with a mean age of 63.1 (SD 6.5) years. One participant was aged <50 years (47 years) but was nevertheless included in the final sample given that this value only slightly deviated from the planned age threshold. Of the 185 participants who completed the study, 131 (70.8%) answered the follow-up questionnaire. A descriptive summary of participants’ sociodemographic characteristics is provided in Table 1.

**Table 1:** Main sociodemographic characteristics of the participants (n=185)^a^.

Characteristic		Participants, n (%)
**Age group (y)**		
	47-60	70 (37.8)
	61-70	83 (44.9)
	70-82	32 (17.3)
**Sex**		
	Female	106 (57.3)
**Duration of hearing difficulties (y)**		
	0-1	54 (29.2)
	2-5	89 (48.1)
	6-10	23 (12.4)
	10-21	19 (10.3)
Presence of tinnitus		65 (35.1)
Previous physician consultation for hearing difficulties		89 (48.1)
Presence of visual problems		134 (72.4)
**Occupation status**		
	Employed	67 (36.2)
**Monthly income**		
	<€1500 (US $1611.21)	62 (33.5)
	€1500-2500 (US $1611.21-$2685.35)	65 (35.1)
	€2500-4000 (US $2685.35-$4296.56)	42 (22.7)
	>€4000 (US $4296.56)	16 (8.6)
**Residential environment**		
	Countryside	2 (1.1)
	Small town	29 (15.7)
	Suburbs	48 (25.9)
	City	106 (57.3)
**Self-estimated noise level at home**		
	Low	67 (36.2)
	Moderate	113 (61.1)
	High	5 (2.7)

^a^These data were acquired during the baseline assessment through a self-report questionnaire.

### Procedure

#### Study Overview

Interested participants contacted the study administrator via email and received extensive written information about the purpose of the project; study design; length of participation and remuneration; possibility to withdraw participation at any time; and data protection, management, and storage. The study design and implementation and data collection, analysis, and storage were conducted in accordance with current literature on ethical considerations in the context of mobile and mHealth apps [34,35]. Security and privacy recommendations were also adhered to. It was clearly stated that a medical diagnostic was not provided in the study. Communication with the participants took place exclusively via email and SMS text message. A pilot study was conducted with a young (aged 23 years), healthy female participant in August 2021 to evaluate the usability and technical functionality of the mobile study.

The web-based study was conducted on the personal mobile phones of the participants to approximate the experience of using an app. Only 3.8% (7/185) of the participants used their computers due to technical difficulties with their smartphones. Data collection was carried out using formr, an open-source web-based application programming interface (API) for the R language that creates automated studies [36]. In formr, different questionnaires and tests (refer to Tables 2 and 3) were linearly chained together as modules of a so-called *run*. A run reproduces the desired design and can be accessed by users through a specific link. The formr software first provides a unique study link to the run, which was shared via email with enrolled participants. Upon accessing this link, participants were assigned a unique visitor session in formr and provided with a second individualized link based on web cookies. The unique visitor session prevented users from providing multiple entries for the same survey. This unique session code enabled the anonymization of the data within formr and, for the duration of the study, was stored in a written coding list, where the participants’ names and session codes were recorded. The customer communication platform Twilio (Twilio Inc) [37] was used to send the individualized study link to the participants through daily SMS text message reminders. Automated SMS text message delivery was initiated via the *external link* module in formr, which uses Representational State Transfer API to connect to Twilio. Representational State Transfer API allows a software program (in this case, formr) to expose functionality and data to other programs (Twilio) in a consistent and secure format, ensuring privacy and data protection. With the individualized link received via SMS text message, participants could perform the study on their own smartphone’s browser. For the 3.8% (7/185) of participants who completed the study on their computer, the daily SMS text messages were sent to their personal mobile phones as reminders. The individualized link was additionally sent via email at the beginning of the study to allow these participants to access the study via their computer browser.

**Table 2:** Assessment of hearing-related features (baseline and longitudinal assessment).

Domain and predictor (assessment tool)			Feature for machine learning
**Participation and handicap**			
	Self-reported hearing difficulties (SSQ^a^ [38])	Speech hearing scale Spatial hearing scale Qualities of hearing scale	
	Consequences of hearing loss (HHIE/A^b^ [39], 2020)	Social consequences of hearing loss scale Emotional consequences of hearing loss scale	
	Social life participation (Social Network Index [40], 2017, adapted from the Department of Psychology, University of Oldenburg)	Social network diversity score Number of people score Number of nets score	
**Attitude toward hearing aids**			
	Hearing aid expectations (ECHO^c^ [38])	Hearing aid expectations (global score)	
	Hearing aid stigma (ALHQ^d^ version 3.0 [41])	Denial of hearing loss scale Negative associations scale Negative coping strategies scale Manual dexterity and vision scale Hearing-related esteem scale	
**Hearing-related personality traits**			
	Noise sensitivity (WNSS^e^ [42], 1997)	Noise sensitivity (global score)	
	Hearing habits (SP-HHQ^f^ [43])	Noise annoyance factor Sound quality factor Noise sensitivity factor Unpredictable sounds factor Openness factor Warm sounds factor Environmental sounds factor	
	Hearing health literacy (HLS-EU-Q16^g^ [44], 2015, with 9 additional internally developed items)	Health literacy (global score)	
**Hearing performance**			
	Hearing performance (SRT^h^; DTT^i^ [45])	SRT mean SRT SD	
	Hearing feedback type	Intermediate (percentage of yellow feedback) Poor (percentage of red feedback)	
**Others**			
	Hearing-related sociodemographic data (sociodemographic questionnaire developed for this study by the authors)	Hearing difficulties—presence Hearing difficulties—duration (years) Hearing difficulties—previous consultation with a health professional Tinnitus—presence Tinnitus—duration (years) Tinnitus—previous consultation with a health professional Motivation to seek professional help before the study Source of motivation to seek professional help before the study Motivation to seek professional help after the study Source of motivation to seek professional help after the study General attitude toward hearing aids	

^a^SSQ: Speech, Spatial, and Qualities of Hearing Scale.

^b^HHIE/A: Hearing Handicap Inventory for the Elderly and Hearing Handicap Inventory for Adults.

^c^ECHO: Expected Consequences of Hearing Aid Ownership.

^d^ALHQ: Attitudes Toward Loss of Hearing Questionnaire.

^e^WNSS: Weinstein Noise Sensitivity Scale.

^f^SP-HHQ: Sound Preference and Hearing Habits Questionnaire.

^g^HLS-EU-Q16: 16-item European Health Literacy Survey Questionnaire.

^h^SRT: speech recognition threshold.

^i^DTT: digit triplet test.

**Table 3:** Assessment of psychological, general health, and sociodemographic features (baseline and longitudinal assessment).

Domain and predictor (assessment tool)			Feature for machine learning
**Personality traits**			
	Big 5 (NEO-FFI^a^ [46])	Neuroticism scale Extraversion scale Openness scale Agreeableness scale Conscientiousness scale	
	Trait anxiety (Geriatric Anxiety Inventory [47], 2016)	Anxiety (global score)	
	Trait depression (Geriatric Depression Scale [48], 1986)	Depression (global score)	
	Optimism and pessimism (The Optimism-Pessimism Scale - 2 [49], 2012)	Optimism (global score)	
	Loneliness (DJG^b^ scale [50], 2013)	Loneliness (global score)	
	Sensory processing sensitivity (HSPS-G^c^ [51])	Ease of excitation scale Sensory threshold scale Esthetic sensitivity scale Hearing scale	
**Attitudes and beliefs**			
	Health locus of control (KKG^d^ [52], 1989; Internal-External Control Belief Scale - 4 [53], 2012)	Internal locus of control scale Society control scale External locus of control scale	
	Attitude toward aging (AAQ^e^ [54], 2007)	Psychosocial scale Physical scale Psychological scale	
	General self-efficacy (GSES^f^ [55], 2003)	General self-efficacy (global score)	
**Mood**			
	Affect (daily questionnaire on affect developed for this study by the authors)	Positive affect pretest mean Positive affect pretest SD Negative affect pretest mean Negative affect pretest SD Positive affect posttest mean Positive affect posttest SD Negative affect posttest mean Negative affect posttest SD	
	Stress (PSS^g^ [56], 2020)	Perceived stress (global score)	
**Cognitive functions**			
	Figural reasoning (BEFKI^h^ [57], 2020)	Figural reasoning (global score)	
	Vocabulary (Vocabulary Test [58], 1992)	Vocabulary (global score)	
	Digital literacy (Technology Readiness – Short scale [59], 2012)	Technology commitment (global score)	
**Others**			
	General health (SF-12^i^ [60])	Physical health score Mental health score	
	General sociodemographic data (sociodemographic questionnaire developed for this study by the authors)	Age Sex Presence of visual problems Educational degree Occupation (retired or working) Weekly working hours Monthly income Relationship status Monthly income of partner Residential environment Household size	

^a^NEO-FFI: Neuroticism-Extraversion-Openness Five Factor Inventory.

^b^DJG: De Jong Gierveld Loneliness Scale.

^c^HSPS-G: Highly Sensitive Person Scale.

^d^KKG: Kontrollüberzeugungen zu Krankheit und Gesundheit (Control Beliefs about Illness and Health).

^e^AAQ: Attitudes to Aging Questionnaire.

^f^GSES: General Self-Efficacy Scale.

^g^PSS: Perceived Stress Scale.

^h^BEFKI: Berlin Test of Fluid and Crystallized Intelligence.

^i^SF-12: 12-item Short-Form Health Survey.

A detailed list of all assessment tools and their references, as well as the derived features for analysis, is provided in Tables 2 and 3. Each assessment tool was implemented as a *survey* in formr. Most of the surveys included in the study were implemented following the paper-and-pencil version that was retrieved from the literature. The surveys that were developed or adapted specifically for this study can be shared upon request. The items assessed can be inferred from the features provided in Tables 2 and 3. Submission of each survey was possible only after all mandatory questions had been answered. After submission of one survey, the study advanced automatically to the following questionnaire or test planned in the formr run. Users were not allowed to go back and modify their answers after submission.

The total assessment time of 8 hours was distributed across the working days of 3 consecutive weeks, with an overall daily active participation of approximately 30 minutes. The study design is detailed in Multimedia Appendix 1. Participants could select the start date of the study to ensure that the assessment could be easily integrated into their personal schedule. The first week (baseline assessment) included 1 measurement time point per day (requiring approximately 20-30 minutes to complete), which could be performed at any time. On the first day, participants received an email with the study link and their unique participant code. After opening the study link on their browser, each participant was given the possibility to read a summary of the data protection conditions in the first page of the study again. Second, they were asked to provide a telephone number and email address, which were then stored in formr and used for the automatic SMS text message reminders. They would then receive an automatic SMS text message with the individualized link to the study, through which they could begin the assessment. From days 2 to 5, participants received an SMS text message with the link to the study at 7 AM, but they had been previously informed that they could perform the tasks at any time during the day. An email reminder was sent at 7 PM in case participants had not accessed the study link by that time.

The second and third week included 2 measurement time points per day of approximately 15 minutes each. The longitudinal assessments were prompted via SMS text message at 7 AM and 7 PM. Participants were instructed to access the study at their earliest convenience after waking up and before going to bed, thus allowing them to accommodate the study to their daily schedules. In the morning, after clicking on the link they received via SMS text message at 7 AM, each participant was first presented with some questions on baseline mood and sleep quality. They were then required to click on a second link embedded in the following survey page that redirected them to the hearing assessment. Finally, participants were asked again to report on their mood after receiving feedback on their hearing performance. In case the participant forgot to access the link and perform the study, an email reminder was sent at 1 PM. If, after the reminder, the participant still did not take part in the study, the session was established as incomplete. The study administrator had to manually allow the participant to move to the next measurement time point (in this case, the evening assessment) within formr. This same assessment scheme was repeated in the evening. In this case, the SMS text message with the link to the study was sent at 7 PM, and the email reminder was sent at 11 PM.

At the end of the study, participants were asked to provide consent to be contacted after 2 months for a voluntary (and nonremunerated) follow-up questionnaire. Those who provided their consent received an email with a link to a single formr survey that required <5 minutes to complete. The short survey consisted of 2 multiple-choice questions and was completed by 70.8% (131/185) of the participants. Individuals were asked to report again on their action to seek professional help following the feedback received during the study and were asked to indicate whether study participation improved their awareness of hearing difficulties.

#### Baseline Assessment

##### Overview

During the baseline assessment, cross-sectional data from a comprehensive set of 25 questionnaires and tests were collected. The questionnaires and tests were distributed on 5 consecutive days to maximize study compliance and avoid priming effects on different questionnaires. The following sections provide a concise summary of the measured predictors (features). We refer to Tables 2 and 3 for a complete list of the assessment tools used in the study. We selected questionnaires and tests that have been previously used in studies investigating their association with hearing help seeking and HA uptake (as cited in the Introduction section and in the Tables 2 and 3). If the tools included in the study had not been previously used in similar literature, we explained our rationale for their selection in the following sections.

##### Assessment of Hearing-Related Features

First, the assessment included self-reports of participation and perceived handicap, focusing on self-reported hearing difficulties, consequences of HL, and social life participation. In addition, attitudes toward HAs were evaluated using questionnaires on HA expectations and stigma. Hearing-related personality traits were also taken into account. Noise sensitivity was measured as a personality trait, which was shown to be related to affect and neurosensory processing [61]. We further assessed hearing habits aiming to gather more information about sound sensitivity and individuals’ sound preference profiles [43]. Finally, hearing health literacy was assessed as well as the ability to search, find, and understand information related to hearing health has been shown to be associated with better self-management of HL [31].

##### Assessment of Psychological, General Health, and Sociodemographic Features

Personality traits (the Big 5 [62]) were shown to be associated with help seeking and HA use and, therefore, were included in the baseline assessment. Anxiety and depression were also measured given their frequently demonstrated associations with HL [4,6], together with loneliness, which is seen as consequence of untreated HL [3]. We further assessed optimism and sensory processing sensitivity, which refers to an individual’s disposition to perceive and process stimuli (including auditory ones) more intensely than the average population [63]. Attitudes and beliefs such as locus of control and self-efficacy were included as well for their association with help seeking and HA use. The belief that HAs are associated with old age and infirmity is often a barrier to HA uptake and use [4]; therefore, attitude toward aging was assessed as well. Perceived stress was measured, too, as high levels of stress that are related to daily life, work, or social situations may boost help-seeking behaviors [3]. General health was assessed given its predictive role for different steps of the HA uptake path [4,6]. For completeness, we also measured cognitive abilities (crystallized and fluid intelligence) despite discordant findings on associations between cognition and HA uptake [4,6,16]. Finally, participants were requested to complete a comprehensive questionnaire on sociodemographics.

#### Longitudinal Assessment of Hearing and Affect

This microlongitudinal assessment accounts for potential daily fluctuations in hearing performance and affect, which might depend on particular daily events and states. The affect questionnaire included 14 items in line with the circumplex model of affect (Posner et al [64]). A total of 8 items were related to negative affect, and 6 items were related to positive affect [65]. The items are listed in Multimedia Appendix 1. The affect questionnaire was presented before and after the hearing test to assess mood at baseline and after receiving feedback on the hearing test, respectively.

Hearing performance was assessed using the digit triplet test (DTT) [45,66] by Hörzentrum Oldenburg gGmbH. This widely used screening instrument [67] measures speech intelligibility in noise by means of the speech recognition threshold (SRT), which indicates the signal-to-noise ratio (SNR; difference between speech and noise level) at which the participant reaches 50% speech intelligibility. SRT measures obtained using the DTT showed high correlations (*r*>0.70) with pure tone average measures while being relatively robust against changes in presentation level [68]. Moreover, the DTT has shown to be robust to ambient noise levels outside of audiometric booth environments [68]. Together with its low linguistic and cognitive demands [68], the DTT appears to be suitable for mobile, remote self-test–based screening of hearing abilities in the older population. Smartphone-based DTT has also shown the potential to provide widespread access to hearing screening in low- and middle-income countries and across different socioeconomic strata [69]. After completing the hearing test, participants received feedback on their performance in the form of a traffic light color, where green indicated good performance (SRT<–7.1 dB SNR), yellow indicated intermediate performance (–7.1≥SRT<–5.1 dB SNR), and red reflected poor performance (SRT≥–5.1 dB SNR) [45,70]. The Multimedia Appendix 1 provides detailed information on the hearing test and Multimedia Appendix 2 provides information on its feedback. Participants performed the hearing test with their personal smartphone and headphones. A total of 1.6% (3/185) of the participants used loudspeakers as headphones were not available to them. Calibration of the hardware equipment was not possible due to the remote assessment. However, SRT estimation is relatively robust against changes in presentation level, and no exact calibration is needed [71]. Moreover, the use of different types or qualities of headphones has shown no impact on test reliability [67,69]. Each hearing test began with a signal adjustment trial meant to set the stimulus at approximately 65 dB sound pressure level. The participant was presented with a digit triplet in noise and asked to “adjust the volume to hear both the digits and the noise clearly.”

#### Outcome Measures

##### Overview

Classification of participants into help seekers and nonseekers was based on self-reports of planned actions to seek professional help for their perceived hearing difficulties and their motivation to seek help. These variables, as retrieved at the end of the study and at follow-up, were chosen as outcome measures for the supervised machine learning (refer to the following sections). This information was also assessed at the beginning of the study. In total, 3 different classifications were considered as outcome measures: action to seek help at study end, action and motivation to seek help at study end, and action to seek help at follow-up. The distribution of participants along the outcome classes considered is summarized in Table 4.

**Table 4:** Absolute classwise frequencies of observations at the 2-month follow-up across the 3 outcomes considered at the end of the study^a^.

	Action at follow-up (n=52, 28.1%), n (%)	No action at follow-up (n=79, 42.7%), n (%)	No follow-up data (n=54, 29.2%), n (%)
Action (n=64, 34.6%)	33 (17.8)	12 (6.5)	19 (10.3)
No action and high motivation (n=47, 25.4%)	12 (6.5)	22 (11.9)	13 (7)
No action and low motivation (n=74, 40%)	7 (3.8)	45 (24.3)	22 (11.9)

^a^The 3 outcomes considered were action to seek help (n=185), action and motivation to seek help (n=185), and action to seek help at follow-up (n=131). In addition, the table provides an overview of those participants who did not complete the follow-up questionnaire.

##### Action to Seek Help

Help seeking (preparation and action [3]) was assessed at study end using the following question: “Given the feedbacks of this study regarding your hearing performance, have you made an appointment with one of the following physicians or a hearing care professional, or are you planning to do so?” (followed by a list of hearing professionals). This variable was used to create two outcome classes: (1) *action* class (64/185, 34.6%)—participants who were planning to seek professional help in the near future or had already made an appointment—and (2) *no action* class (121/185, 65.4%)—participants not ready to take action who did not plan to consult a hearing health professional in the future.

##### Action and Motivation to Seek Help

A second outcome measure was taken into account to further differentiate the *no action* class to provide further insights for the design of targeted recommendations in an mHealth hearing app. Information on readiness to take action was combined with the reported motivation to seek help at the end of the study. Motivation was assessed through the following question: “How motivated are you at the moment to seek help regarding your hearing problems?” (1=not motivated at all; 7=very strongly motivated). The answer spectrum was binarized by means of median split to create the following outcome classes: (1) *no action and high motivation* class (47/185, 25.4%)—participants not ready to take action with high motivation who might particularly benefit from personalized and tailored recommendations, (2) *no action and low motivation* class (74/185, 40%)—participants not ready to take action who reported low motivation to seek help, and (3) *action* class (64/185, 34.6%)—participants ready to take action regardless of their motivation level (this class was not further divided with respect to motivation as this would not result in different recommendations. Moreover, data exploration revealed that only 11% (7/64) of the individuals in this category reported low motivation).

##### Action to Seek Help at Follow-Up

The voluntary follow-up questionnaire (completed by 131/185, 70.8% of the participants) included a question on the intention or action to seek help following the feedback received during the study. The answer range (4 multiple-choices) was binarized to achieve a class allocation comparable to that of the first outcome measure: (1) *action at follow-up* class (52/131, 39.7%)—participants who reported having completed an appointment with a hearing professional, who had an appointment scheduled but not completed, or who were planning to seek help in the near future; and (2) *no action at follow-up* class (79/131, 60.3%)—participants who did not plan to consult a hearing health professional.

### Statistical Analysis

#### Data Preprocessing

Data analysis was performed using the R software (R Foundation for Statistical Computing) [72]. Raw data from all questionnaires were imported from the web-platform formr to the R environment using the dedicated *formr* package [73]. For each questionnaire or test presented at baseline, global scores were computed and considered as features. If both global scores and scale scores were available for a given assessment tool, only the scale scores were retained if considered differentially relevant for the outcome. Hearing test results were sent via email to the investigator from the researchers of Hörzentrum Oldenburg and imported into R as .eml files. The performance feedback category (green, yellow, and red) was additionally extracted and stored for each raw SRT result. Due to the particular implementation of the study in formr, participants could perform the hearing test more than once at each measurement time point. Whenever this happened, only the last SRT result at a given time point was kept for analysis. This led to a removal of 3.9% of the raw SRT results. The longitudinal data on daily mood and hearing performance were summarized into individual means and SDs. The summarized longitudinal data were merged with the cross-sectional data, resulting in a wide-format data frame including 83 features. Tables 2 and 3 provide a complete list of the features considered for analysis.

Completion rate for the baseline questionnaire was 100% (185/185), whereas there were missing data for the longitudinal measures of hearing performance and affect. A complete set of 20 SRT results was collected for 43.8% (81/185) of the participants, whereas at least 15 SRT results were obtained in 95.1% (176/185) of cases. Missing hearing data at a specific time point were considered not available (NA). Where an SRT result was missing, the respective feedback and measures of affect at the posttest time point were established as missing as well (NA). Through visual inspection of the individual SRT distributions, some specific outlier patterns were identified. A total of 7.6% (14/185) of the participants showed a much larger SRT result at the first measurement, which qualified as an outlier following the IQR rule. These large SRT values (indicating poor performance) were considered to be caused by misunderstanding of the hearing test instructions and, therefore, were established as NA. However, the respective feedback category and measures of affect where not established as NA. This is because, despite the unreliable SRT value, participants’ mood could still have been affected by the feedback received. With respect to daily affect measures, 1.1% (2/185) of the participants provided no data at measures of posttest affect such that summary measures could not be computed. In these cases, the mean imputation technique was applied—the sample mean and sample variance for negative and positive affect at the posttest time point were imputed to replace missing values.

#### Machine Learning

##### Overview

The data were fed into 3 machine learning algorithms for supervised classification. We chose naïve Bayes (NB), random forest (RF), and support vector machine (SVM) among other classifiers to cover a wide range of model complexity (from simple models such as NB to more complex and nonlinear ones such as SVM). The algorithms were implemented in R using the *mlr* package [74] following the approaches described in the work by Rhys [75] and Bischl et al [76]. Given the presence of 3 different outcome measures, the same analysis steps were carried out in parallel for each outcome with a slight difference in the input features included in the analysis. For the first outcome (*action to seek help*), data on motivation at the prestudy time point were kept in the feature space, whereas motivation to seek help at the end of the study was removed. The same applied to the third outcome (*action to seek help at follow-up*). Differently, for the second outcome (*action and motivation to seek help*), all data on motivation at the pre- and poststudy time points were removed from the feature space. Due to the relatively small data set and imbalanced classes, we chose not to split the data into training and test sets but to use CV instead. CV divides the training set into *k* equally sized parts and considers the *k*th part as a test set and the *k* – 1 part as a training set at each iteration. Model results are then averaged across all iterations. The implementation details of the 3 algorithms are summarized next.

##### NB Classifier

This algorithm uses the Bayes rule to predict the probability of an observation belonging to one of the outcome classes given its discriminant function values. Given the prior probability, the likelihood, and the evidence for each observation, the relative posterior probability for each class is computed. The single observation is then assigned to the class with the highest relative posterior probability [75]. The 2 strong assumptions made by NB algorithms are the normal distribution of continuous features (or predictors) and the independence of these features. Model performance will suffer in case of violation of these assumptions [75]. In this implementation, after training the algorithm, repeated 10-fold CV was used to evaluate the model’s performance. A stepwise approach was used to select the appropriate number of CV repetitions necessary to achieve accurate and stable performance estimates (50, 100, 150, and 300 CV repetitions).

##### RF Classifier

Tree-based methods use recursive binary splitting to stratify the features’ space in smaller, nonoverlapping regions used for classification. At each iteration of the tree-building process, the algorithm selects among all features the one that best splits the data into 2 branches according to a specific question or rule (node) [75]. The process iterates until a stopping criterion is met and final regions (leaves) are identified. In a classification problem, the mode of the training data within a region is used for prediction—each observation is classified to the majority class within the leaf to which it belongs [77]. Trees are easy to interpret, but they lack predictive power as they tend to overfit the training data. Approaches such as RF can be used to improve prediction accuracy. RF is a nonlinear method that involves the generation of multiple uncorrelated trees from different bootstrapped training sets obtained through sampling with replacement from the original data. The final predicted outcome is retrieved from aggregating the prediction of all built trees and selecting the most frequent or modal prediction [75,77]. This algorithm requires the tuning of a set of hyperparameters that control the learning process and are selected (or tuned) by the algorithm to obtain the best performance. The hyperparameters shown in Textbox 1 were considered.

**Hyperparameters for RF classifier.**

***Ntree***

The number of trees to include. This value is usually fixed at a computationally reasonable value rather than tuned [75]. Ntree was set to 800.

***Mtry***

The number of features to randomly sample at each time. A popular value is given by √p (where p=the number of predictors) [78]. Different search spaces were explored, with Mtry ranging between 1 and 15.

***Nodesize***

The minimum number of cases to be included in a leaf. Different search spaces were explored, with Nodesize ranging between 1 and 20.

Tuning the algorithm and finding the best hyperparameter combination requires the definition of an optimization algorithm, or search strategy, and evaluation method. We used grid search with 10-fold CV resampling. To evaluate model performance, nested CV was applied. In this approach, an inner loop tunes the hyperparameters, and an outer loop evaluates a wrapped learner, which comprises the classification task, the learner type (RF), and the hyperparameter tuning process. In this case, a 5-fold CV was applied as an outer resampling strategy.

##### SVM Classifier

The SVM algorithm iteratively identifies a hyperplane that separates labeled classes also in case of nonlinear data distributions. It does so by adding an extra dimension to the data, which is found through the kernel function, a mathematical transformation of the data. The hyperplane is defined as a surface that has 1 dimension less than the number of variables in the data set. The position of the hyperplane depends on the position of the support vectors, which are training set cases that define the class boundaries [75]. The optimal hyperplane is found by maximizing its margin, which is the region around the hyperplane that touches the fewest training observations. In fact, the distance from a training case to the margin can be viewed as a measure of the correctness of its classification [77]. In case the algorithm needs to separate >2 classes, several models are built and compared to find the one that best predicts new data. SVMs are computationally expensive but tend to perform very well on a variety of tasks conducted on nonlinearly separable classes. In addition, the algorithm has the advantage of making no assumptions on the features’ distributional properties [75,77]. Similar to RF, SVM requires hyperparameter tuning. The hyperparameters in Textbox 2 were considered.

**Hyperparameters for SVM classifier.**

**Kernel**

The type of kernel function used to identify the hyperplane. Polynomial, radial, and sigmoid functions were included in the search space [75].

**Degree**

The shape of the decision boundary (in case of a polynomial kernel). The search space was limited to values from 1 to 5 to avoid the risk of overfitting [75].

**Cost**

The penalty for having cases fall inside the margin. It is recommended to tune both cost and gamma (refer to the next point) on the logarithmic scale [79], and a popular search space for cost is from 2^–5^ to 2^15^ [80].

**Gamma**

Influence of each case on the hyperplane. This hyperparameter search space was set to the popular range of 2^–15^ to 2^3^ [80].

Nested CV was used as previously described for RF. An inner resampling loop (with 10-fold CV) was applied for hyperparameter tuning, and an outer loop (with 5-fold CV) was applied for performance evaluation.

##### Classification Performance Metrics

The algorithms were evaluated in terms of prediction accuracy on the test set, which indicated the overall proportion of cases correctly classified by the model as compared to the observed outcome. However, class imbalance in the sample can negatively impact prediction accuracy, reducing its informativeness as a performance measure [81,82]. The Matthews correlation coefficient (MCC; *yardstick* package [83]) was additionally taken into account to evaluate model performance. This coefficient improves over accuracy measures in case of imbalanced data sets [82,84] and can take values from –1 to 1, with 1 indicating perfect prediction, 0 indicating chance prediction, and –1 indicating inverse prediction. As an additional metric, we computed the confusion matrix (calculateConfusionMatrix(), *mlr* package). Its output provides the absolute number and the proportion of correct model predictions and misclassifications for each outcome class. For binary outcomes, the confusion matrix allows for the estimation of model sensitivity (ie, accurately identifying help seekers) and specificity (ie, accurately identifying nonseekers). In this study, obtaining high specificity was of particular interest in the context of an mHealth hearing app. Indeed, individuals with HL who are not prone to seeking help are the main target population for tailored treatment recommendations and counseling.

#### Feature Importance

After identifying the best-performing machine learning algorithm, feature importance was considered. Each feature receives a coefficient of importance that indicates its contribution to model performance regardless of the type of relationship (direction and linearity) between the feature and the outcome [85]. In RF, feature importance is model dependent and indicates how much the feature contributes in reducing node impurity. Importance values were retrieved using the function getFeatureImportance() (*mlr* package) applied on the RF model trained using the tuned hyperparameters. These importance results have the advantage of being inherent to the model and closely tied to its performance [86]. Conversely, there are no model-specific importance metrics available for NB and SVM. For these algorithms, the importance value assigned to each feature corresponds to the area under the receiver operating characteristic curve, which is computed from sensitivity and specificity measures [86]. The function varImp() from the *caret* package [87] was applied on the model trained using the function train() (*caret* package) after ensuring comparable performance with that of the same model previously trained on the *mlr* package.

Features with higher importance ranking were considered for inclusion in the profiling algorithm as they represented the most relevant predictors for telling apart help seekers and nonseekers. No statistical criterion exists to determine which importance value threshold should be used to retrieve relevant features. Hence, 3 threshold values were inspected (the first 10, 15, and 20 features in their importance ranking order) and evaluated in terms of predictive accuracy and interpretability. The classification accuracy of these 3 feature sets was assessed on the outcome data obtained at follow-up. For this analysis, the data set was reduced to 131 participants who completed the follow-up questionnaire. The important features were fed into the best-performing machine learning algorithm from the previous step.

### Ethical Considerations

The study plan and data management have been approved by the Research Ethics Committee of the Carl von Ossietzky Universität Oldenburg (08.09.2021; EK/2020/020-01). The study supported the autonomy of participants through extensive informed consent, which was given both as a separate written document before enrollment and within the formr survey framework. Debriefing was included, and participants were invited to provide feedback at the end of the study. No direct risks associated with the study design were identified, and privacy risks were accounted for through appropriate data management and data protection concepts for all software and platforms used. Personal information collected during the study was pseudoanonymized using a written coding list stored in a closed locker accessible only to the study administrator. This coding list was destroyed at the end of data collection; therefore, the data have been completely anonymized since. Data collection took place between September 2021 and September 2022. Participants were remunerated with €10 (US $10.74) per hour. A further incentive for study participation was that participants received written feedback on their daily hearing test results.

## Results

### Machine Learning Classification Performance

#### Predicting Action to Seek Help

**Figure 1:**
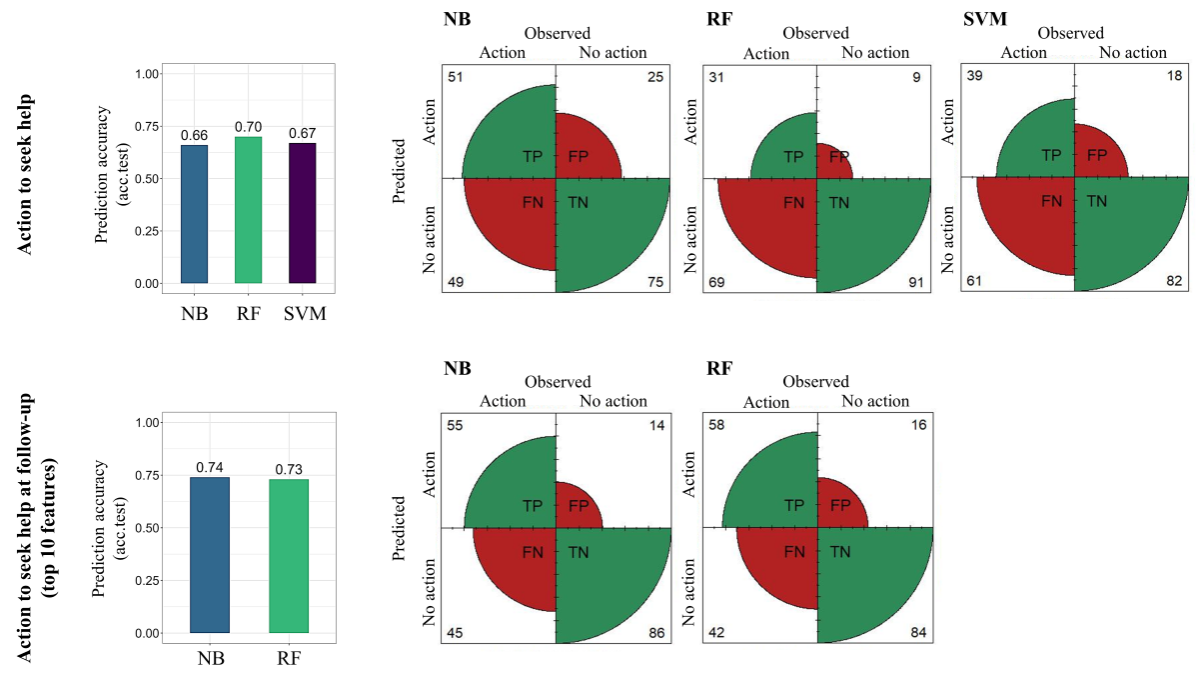
Visualization of the model-specific performances (left) and relative confusion matrices (right) for the outcomes action to seek help (first row) measured at study end (n=185) and action to seek help at follow-up (second row) considering the set of top 10 most important features (n=131). Acc.test: prediction accuracy on the test set; FN: false negative; FP: false positive; NB: naïve Bayes; RF: random forest; SVM: support vector machine; TN: true negative; TP: true positive.

A summary of the model-specific classification accuracy for the first outcome (*action to seek help*) is provided in Table 5. The 3 algorithms showed similar overall performance accuracy estimates on the test set, correctly classifying approximately 65.9% (122/185) to 70.3% (130/185) of the cases in the full data set (n=185). RF was the best-performing algorithm with an accuracy of 70.3% (130/185) and an MCC of 0.28, indicating that the model’s prediction improved to approximately 20% over chance. By inspecting the confusion matrix (Figure 1), we observed that RF shows high specificity, correctly classifying 90.9% (110/121) of the cases belonging to the *no action* class. The NB classifier (10-fold CV repeated 50 times) showed the best sensitivity compared with the competing algorithms, with 51% (33/64) of cases in the *action* class being correctly classified. RF and NB were selected for feature importance analyses given the good predictive performance and high specificity of RF as well as the high sensitivity of NB.

**Table 5:** Model-specific overall performance and class-specific classification accuracy rates for the first outcome, *action to seek help*, measured at study end^a^.

Hyperparameters								Overall performance measures					Class-specific classification accuracy		
Model and parameter			Parameter space		Tuned value		Test accuracy			MCC^b^		Action (n=64)			No action (n=121)
**NB^c^**															
	—^d^	—		—		0.66			0.26		0.51			0.75	
**RF^a^**															
	Ntree	800		800		0.70			0.28		0.31			0.91	
	Mtry	(5, 15)		13		—			—		—			—	
	Nodesize	(1, 5)		1		—			—		—			—	
**SVM^f^**															
	Kernel	—		Radial		0.67			0.23		0.39			0.82	
	Degree	(1, 5)		4		—			—		—			—	
	Cost	(2^–5^, 2^15^)		0.01		—			—		—			—	
	Gamma	(2^–^^15^, 2^3^)		69.1		—			—		—			—	

^a^Results are based on the full data set (n=185). For random forest and support vector machine, the table additionally shows the hyperparameter search space used in the resampling procedure and the tuned values used for model training and feature importance analysis. The selected naïve Bayes model used 10-fold cross-validation repeated 50 times.

^b^MCC: Matthews correlation coefficient.

^c^NB: naïve Bayes.

^d^Not applicable.

^e^RF: random forest.

^f^SVM: support vector machine.

#### Predicting Action and Motivation to Seek Help

Table 6 summarizes the classification performance with respect to the second outcome (*action and motivation to seek help*), which includes 3 classes (refer to the previous section). RF provided the highest accuracy with 55.1% (102/185) and an MCC of 0.30 as compared to NB (10-fold CV repeated 100 times) and SVM. However, the confusion matrix revealed that none of the 3 models was able to adequately tell apart individuals within the no action class who differed with respect to high versus low motivation. All algorithms could only correctly classify 2% (1/47) to 25% (12/47) of cases in the no action and high motivation class. Potentially, an improvement in classification accuracy could be achieved using a larger data set in which the classes are better balanced and with a more reliable and elaborate measure of the participants’ motivation to seek help. In view of these limitations, the second outcome was not considered for feature importance analysis.

**Table 6:** Model-specific overall performance and class-specific classification accuracy rates for the second outcome *action and motivation to seek help* measured at study end^a^.

Hyperparameters					Overall performance measures			Class-specific classification accuracy		
Model and parameter		Parameter space	Tuned value	Test accuracy		MCC^b^	Action (n=64)		No action and low motivation (n=74)	No action and high motivation (n=47)
**NB^c^**										
	—^d^	—	—	0.50		0.22	0.47		0.67	0.25
**RF^e^**										
	Ntree	800	—	0.55		0.30	0.55		0.80	0.09
	Mtry	(8, 10)	—	—		—	—		—	—
	Nodesize	(3, 15)	800	—		—	—		—	—
**SVM^f^**										
	Kernel	—	Sigmoid	0.49		0.20	0.53		0.76	0.02
	Degree	(1, 5)	1	—		—	—		—	—
	Cost	(2^–^^5^, 2^15^)	323	—		—	—		—	—
	Gamma	(2^–^^15^, 2^3^)	3.05 × 10^5^	—		—	—		—	—

^a^Results are based on the full data set (n=185). For random forest and support vector machine, the table additionally shows the hyperparameter search space used in the resampling procedure and the tuned values used for model training and feature importance analysis. The selected naïve Bayes model used 10-fold cross-validation repeated 100 times.

^b^MCC: Matthews correlation coefficient.

^c^NB: naïve Bayes.

^d^Not applicable.

^e^RF: random forest.

^f^SVM: support vector machine.

### Feature Importance

#### Predicting Action and Motivation to Seek Help at Follow-Up

Feature importance was analyzed based on the RF and NB algorithms predicting the first outcome, *action to seek help* at study end on the full data set (n=185). Each importance value signifies the feature’s contribution to the model’s performance. However, as detailed in the Methods section, RF and NB models calculate these coefficients differently. Consequently, ranking values were used. In both models, the 83 features were initially ranked in descending order based on their importance values. Features with higher importance rankings were mostly relevant in predicting help-seeking behavior. Three sets of features among the most important ones were taken into account for subsequent analysis: (1) the top 10 features indicated by the 2 models, resulting in a total of 12 best features; (2) the top 15 features indicated by the 2 models, resulting in a total of 19 best features; and (3) the top 20 features indicated by the 2 models, resulting in a total of 28 best features.

**Figure 2:**
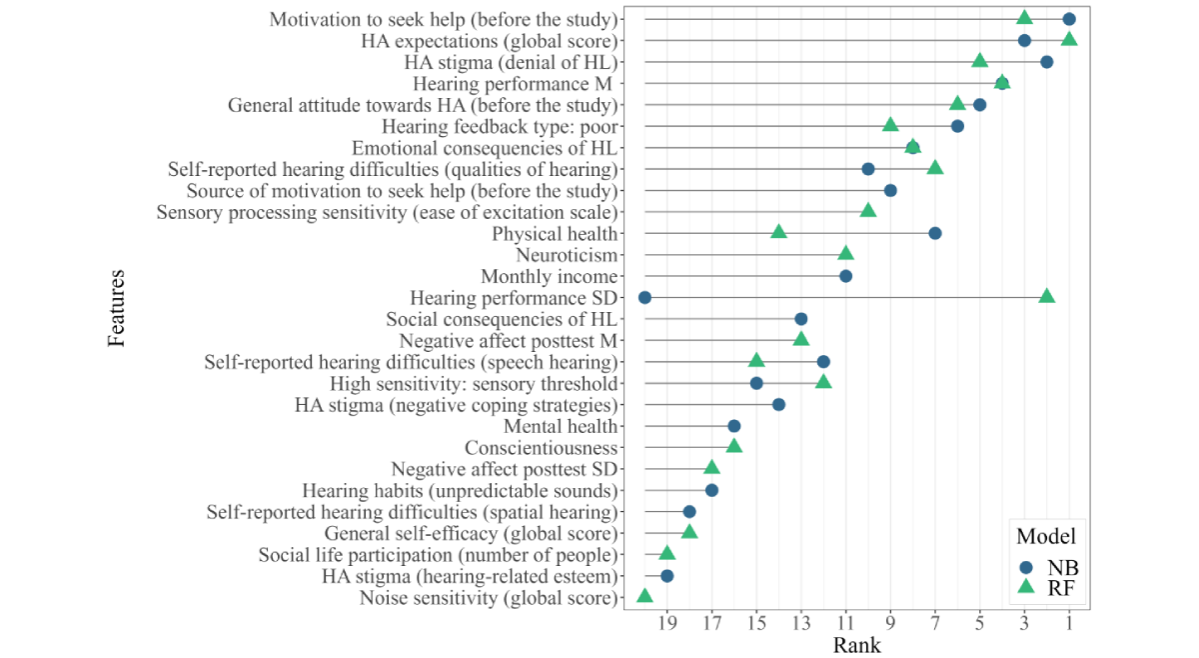
Ranking of the most important features to predict action to seek help at study end. Importance rankings are shown for the 20 most important features for the 2 models (naïve Bayes [NB] and random forest [RF]) trained on the full data set (n=185), as summarized in Table 5. A total of 28 features are arranged on the y-axis with respect to their average ranking between the 2 models. HA: hearing aid; HL: hearing loss; M: mean.

Figure 2 shows all 28 features with their importance ranking values originating from the RF and NB models. The specific importance values for each feature are provided in Multimedia Appendix 3. Next, the 3 sets of features (top 10, 15, and 20 features) were evaluated for their predictive performance and classification accuracy on the reduced data set of 131 participants who completed the follow-up questionnaire. NB and RF were trained on the 3 different feature sets for predicting the *action to seek help at follow-up*. The results are summarized in Table 7. They show that all feature sets provided good predictive performance and that the NB algorithm outperformed RF, with an overall accuracy ranging between 73.3% (96/131) and 74.8% (98/131) and an MCC between 0.43 and 0.47. Class-specific classification accuracy was comparable between NB and RF, with the action class correctly classified in 52% (27/52) to 63% (33/52) of the cases and the *no action* class correctly classified in 82% (65/79) to 86% (68/79) of cases. As can be seen from the confusion matrix in Figure 1, sensitivity and specificity measures were comparable for both algorithms predicting action to seek help at follow-up using the set of top 10 most important features. Sensitivity ranged from 55% (NB) to 58% (RF), and specificity ranged from 84% (RF) to 65% (NB).

**Table 7:** Model-specific overall performance and class-specific classification accuracy rates for the outcome *action to seek help at follow-up*^a^.

Hyperparameters							Overall performance measures					Class-specific classification accuracy		
Model and parameter				Parameter space		Test accuracy			MCC^b^		Action (n=52)			No action (n=79)
**Top 10 features (total=12)**														
	**NB^c^**													
		—^d^	—		0.74			0.44		0.55			0.86	
	**RF^e^**													
		Ntree	800		0.73			0.43		0.58			0.84	
		Mtry	(1, 4)		—			—		—			—	
		Nodesize	(1, 5)		—			—		—			—	
**Top 15 features (total=19)**														
	**NB**													
		—	—		0.73			0.43		0.58			0.83	
	**RF**													
		Ntree	800		0.73			0.41		0.56			0.84	
		Mtry	(1, 10)		—			—		—			—	
		Nodesize	(1, 5)		—			—		—			—	
**Top 20 features (total=28)**														
	**NB**													
		—	—		0.75			0.47		0.63			0.83	
	**RF**													
		Ntree	800		0.70			0.36		0.52			0.82	
		Mtry	(1, 5)		—			—		—			—	
		Nodesize	(1, 5)		—			—		—			—	

^a^Results are based on the reduced follow-up data set (n=131). The table shows the results for 3 sets of features (10, 15, and 20 most important features for the 2 models). For naïve Bayes, 10-fold cross-validation repeated 50 times was used. For random forest, the table additionally shows the hyperparameter search space used in the resampling procedure. In contrast to the previous results (Tables 5 and 6), the tuned hyperparameters were not retrieved as no importance analysis followed.

^b^MCC: Matthews correlation coefficient.

^c^NB: naïve Bayes.

^d^Not applicable.

^e^RF: random forest.

#### Important Features

Textbox 3 provides a brief description of 12 features ranked among the most important (previously named as the top 10 features for the 2 models) in the prediction of *action to seek help at follow-up*.

**12 Features ranked among the most important in the prediction of action to seek help at follow-up.**

- *Motivation to seek help* and *source* of this motivation at the beginning of the study
- Individuals’ attitude toward and expectations regarding HAs, including *general attitude toward HAs*; *expectations regarding HAs*, as measured using the global score of the Expected Consequences of Hearing Aid Ownership questionnaire [38], which assesses positive and negative expectations regarding HAs, expected services and costs, and assumptions about change in the personal image in case of HA use; and *stigma toward HAs*, as measured using the Denial of Hearing Loss scale of the Attitudes Toward Loss of Hearing Questionnaire [41], which assesses acceptance of HAs and acknowledgment of HL
- *Hearing performance* (mean SRT and its variability as measured using the DTT [45,66]) and *percentage of negative feedback received* (indicating poor performance)
- Perceived consequences of HL, including *emotional consequences of HL*, as measured using the corresponding subscale of the Hearing Handicap Inventory questionnaire [39], and *self-reported hearing difficulties*, as measured using the qualities of hearing subscale of the Speech, Spatial, and Qualities of Hearing Scale [38], which addresses recognition, perceived clarity and naturalness of everyday sounds, and listening effort experienced in different hearing contexts
- *High sensory sensitivity personality*, as assessed through the ease of excitation subscale of the Highly Sensitive Person Scale questionnaire [51], which assesses emotional reactivity to physiological stimuli
- Reported *physical health*, measured using the corresponding items of the 12-item Short-Form Health Survey [60]

As outlined in the Methods section, ranking values up to 10 were arbitrarily chosen to identify the most important features. The following two features had slightly lower importance values (and received a ranking value of 11) but might provide further insights into targeted counseling in an mHealth hearing app: (1) *neuroticism*, which refers to a predisposition to experiencing negative emotions [2] and was assessed through the corresponding items of the Neuroticism-Extraversion-Openness Five-Factor Inventory questionnaire [46]; and (2) *monthly income*, which was categorized using 3 cutoff values (<€1500 [<US $1611.21], €1500-€2500 [US $1611.11-$2685.35], €2500-€4000 [US $2685.35-$4296.56], and >€4000 [>US $4296.56]).

## Discussion

### Principal Findings

#### Overview

This study contributes to the identification of individuals’ hearing-related, psychological, and general health–related traits that predict the readiness to seek professional help for HL. Cross-sectional and longitudinal data were collected in a comprehensive mobile study. Potential users of a future mHealth hearing app, namely, individuals with subjective hearing difficulties, were classified into help seekers and nonseekers by means of supervised machine learning algorithms. The trait measures used in this study were collected from previous literature investigating health care seeking, particularly in the audiological domain. From these, we derived a comprehensive set of 83 features to be used for prediction and profiling. The 3 algorithms taken into account (NB, RF, and SVM) accurately predicted help-seeking behavior at the end of the study in 65.9% (122/185) to 70.3% (130/185) of cases. In particular, the RF algorithm achieved high specificity, meaning that it was most successful in identifying individuals who might not intend to seek professional help. By selecting a subset of important traits revealed by our empirical feature importance analyses to predict hearing help seeking, the prediction accuracy for action to seek help at the 2-month follow-up reached 74.8% (98/131). This study identified the following features to be most important in the prediction of help-seeking behavior: perceived consequences of HL in daily life, motivation to seek help, attitudes toward HAs, sensory sensitivity, neuroticism, physical health, and income. We conclude that these individual characteristics should be assessed in a profiling module that could complement the main auditory assessment module for hearing screening of existing or future mHealth apps. The degree of HL but, importantly, also its day-to-day variability were among the most important predictors, suggesting the need to perform repeated hearing assessments, which could be prompted by the app at different times of the day. To streamline the implementation of the profiling module in a mobile app, the questionnaires and subscales used to measure these important features should undergo item selection analysis to derive simple and short yet reliable and valid scales. By incorporating a selected machine learning algorithm (RF), the app can profile users into help seekers or nonseekers based on the data collected through this short questionnaire battery. This information would complement the audiological data gathered through existing hearing screening or diagnostic tests, providing an informative user profile. The derived profile would guide the app in selecting the appropriate set of recommendations, optimizing an intervention on help-seeking behavior where needed. The results of this study will also provide suggestions for the design of such targeted treatment recommendations. Ultimately, our aim was to provide clinicians and mHealth app developers with relevant knowledge to promote hearing health by encouraging the uptake of hearing health care services and HAs when needed.

#### Best Machine Learning Model to Predict Help Seeking and Categorize Individuals Into Help Seekers Versus Nonseekers

In total, 3 machine learning classifiers correctly predicted *action to seek help* at study end in 65.9% (122/185) to 70.3% (130/185) of cases, clearly improving over chance prediction. This is a promising result considering the complexity of the prediction outcome. As discussed previously, several individual factors can influence the decision to seek hearing health care services, and there can be discrepancy among contemplating, planning, and taking concrete action [3]. RF showed the best prediction accuracy and high specificity, whereas NB showed the highest sensitivity. When predicting *action to seek help at follow-up* using the selected important features, the performance of the RF and NB models improved up to 75% despite the smaller data set (n=131). NB showed higher predictive performance for this outcome. Overall, all models exhibited high specificity (ranging from 75% to 86%) and comparatively low sensitivity (31% to 58%). This could be attributed to the fact that the *action* class encompassed individuals who had sought professional help as well as those who were only considering taking action. RF showed high accuracy in identifying the *no action* class both at study end and at follow-up and, therefore, can be considered the best-performing algorithm in this framework. Accurate identification of nonseekers is the most relevant performance outcome in an mHealth app to design targeted recommendations. Indeed, the envisioned profiling algorithm should be a system with high specificity that motivates and promotes help seeking, especially in those cases in which users would not spontaneously take action.

#### Most Relevant Hearing-Related and Psychological Features to Classify Individuals Into Help Seekers Versus Nonseekers

Hearing performance appears to be one of the most important features to predict help seeking. The association between degree of HL and help seeking, as well as HA uptake, is well established in the literature [4,9,26,28,29]. These results also highlight—to our knowledge, for the first time in the literature—the predictive role of intraindividual fluctuations in hearing performance, emphasizing the need to move beyond the traditional view of hearing as a stable neurosensory process [88]. The implementation of repeated daily measurements of hearing performance provides further insights on the impact of HL on the individual’s everyday life. In line with this, feature importance findings emphasize the relevance of self-reports on the consequences of HL. The assessment should consider self-reported listening effort in different contexts as well as perceived handicap and emotional consequences of HL. Indeed, individuals who report greater negative impact of HL in their lives are more prone to seek help and later uptake HAs [9,27]. Individuals’ self-awareness of HL can be validated or improved by providing repeated feedback on hearing performance in an mHealth hearing app. As observed at the follow-up survey, 85.5% (112/131) of participants reported increased awareness of their hearing abilities after receiving repeated feedback during the study. Finally, according to these results and previous findings [26,27,29], investigating stigma, attitude, and expectations regarding HAs informs on individuals’ readiness to seek help as well as later uptake of an HA. Stigma and negative stereotypes related to HAs may deter individuals from seeking help and can represent a barrier to HA use [7,28].

Audiological factors emerged as the most important features. Nevertheless, other general health and psychological factors were also relevant in the prediction of help seeking. In this study, physical health was an important predictor for help seeking, although the evidence on this relationship is discordant [26]. While people with better self-reported health were more likely to seek help [4,27], HA uptake was predicted by poor self-reported health [6]. Other important factors were related to the personality traits of sensory sensitivity and neuroticism. Individuals characterized by high sensitivity to sensory stimuli [63] and emotionally instable personality traits seem to perceive increased psychological discomfort following HL even in the presence of effective HA treatment [2]. Finally, income emerged as another important predictive feature. This is in line with evidence suggesting that higher socioeconomic status [9], higher income or pension earnings [3,27], and access to financial support [26] promote HA uptake.

#### Feature Importance Measures Can Inform the Design of Targeted Recommendations for Users of a Future mHealth Hearing App

By assessing and analyzing the aforementioned hearing-related and psychological traits, the algorithm developed in this study aims to profile the user as help seeker or nonseeker. Completing this profile with complementary audiological test results, an informative picture of the user can be derived. Using this profile, clinical experts and intervention app designers could propose different sets of recommendations to assist individuals in their decision-making process in a targeted manner. We propose to first differentiate between profiled help seekers and nonseekers, where the former should receive simple and straightforward recommendations only depending on their hearing status. For nonseekers, there is a need to design more specific and targeted recommendations based on information about the relevant characteristics to predict HA seeking. Users who were profiled as determined help seekers could receive clear and concise guidance on the hearing care they need. Those among them who should uptake a hearing device (given their audiological outcome) could benefit from additional information on available hearing care services and professionals to facilitate faster HA adoption rates. This would facilitate individuals’ perceived competence and autonomy, which are important predictors of hearing health–seeking behavior [89]. On the other hand, users with HL who are profiled as nonseekers should receive more elaborate, targeted recommendations to motivate and promote access to hearing care services. Recommendations for nonseekers should be further differentiated and designed depending on their perceived consequences of HL; attitudes toward HAs; sensory sensitivity; neuroticism; and, potentially, income. These recommendations could act as an intervention on modifiable predictive features such as self-recognition of HL and attitude toward HAs. For example, users profiled as nonseekers with good awareness and self-recognition of HL but negative expectations regarding HAs and low income should receive a different set of recommendations than nonseekers with low self-awareness and high neuroticism.

Where HL is detected, the mHealth app could prompt repeated testing on different days and at different times of the day and provide individual feedback on the performance compared to normative data. More detailed feedback on daily hearing performance could improve awareness of the hearing deficit. The app could also inform the user of the risks of an untreated HL and the benefits of early intervention through HAs. Indeed, it has been shown that individuals are more likely to positively change their behavior when provided with actionable and meaningful information on their health status [90]. Where there is a need to promote positive attitudes toward HAs, information could be provided on the wide range of devices available as well as examples of successful peer cases. Knowledge of accessible financial support for HAs by insurance companies could additionally promote HA uptake given the predictive role of income. Furthermore, an implemented HA simulator in an mHealth hearing app could offer possibilities to experience improved listening conditions and promote positive expectations regarding an HA. Elaborate information on HA technologies, such as the benefits of noise control and noise reduction algorithms, could promote help seeking by fostering the knowledge of individuals who are more sensitive to environmental noise (high sensory sensitivity trait). The effectiveness of such recommendations could be further increased through targeting or tailoring communication. Targeted messages are designed for a specific population, whereas tailored communication is individualized to the person and has been shown to be most effective in promoting health behavior change [91]. Indeed, messages that are congruent with the personality traits of the audience are more positively evaluated and persuasive and generate more interest [92]. The predictive role of neuroticism for help-seeking behavior can be considered for efficient communication both in the context of an mHealth hearing app and in clinical counseling. Individuals with a high neuroticism trait are more susceptible to perceived disease [93] and are drawn to action through motives of safety and security [92]. For example, recommendations that target profiled nonseekers with low expectations regarding HAs can be differentiated depending on personality traits. To promote positive HA expectations in individuals with high sensory sensitivity traits, recommendations could focus on the benefits of HAs related to noise control and noise suppression. However, such recommendations may not be effective for people who do not have this high sensitivity trait and who, for example, score high on neuroticism. Instead, they might be more convinced by recommendations that emphasize the risks of an untreated HL and the benefits of an early intervention through HAs.

### Limitations and Future Directions

The predictive performance of the machine learning classifiers could be improved in future studies using a larger data set and more balanced classes. Classification accuracy could be further improved by including additional objective measures to complement participant self-reports. Continuous psychophysiological measurements (eg, heart rate variability) could be included as further predictive features. This information could complement the longitudinal assessment of affect and better characterize potential changes in arousal before and after the completion of the auditory measurements. Note that multicollinearity as a potential statistical limitation was ruled out (the correlation plots are available in Multimedia Appendix 4). Future studies might also benefit from a longer follow-up period to properly capture those individuals who took more time to take action to seek help. In this study, measuring help seeking 2 months after the end of the study provided a more valid measure of participants’ behavior. For example, of the participants who were categorized as nonseekers at study end, 3.8% (7/185) reported having made an appointment with a hearing professional at follow-up and 6.5% (12/185) were planning to do so in the near future. Another limitation that affects the generalizability of the findings is the specific sample included in this study—older individuals living in Germany, using a smartphone in their daily life, mainly coming from big cities (106/185, 57.3%), and having a monthly income above the national average net salary (€2500 [US $2685.35]) in 31.4% (58/185) of cases. The conclusions about relevant personal factors for predicting hearing help seeking may not be generalizable to people with HL in different socioeconomic situations. For example, socioeconomic factors were found to be the major limiting factor in seeking help for hearing difficulties in a South African periurban community [94]. To address this sociodemographic limitation, future studies may consider alternative recruitment strategies to achieve a more diverse sociodemographic sample. These findings may also not generalize to different age groups. For example, when considering young adults with HL, other personal characteristics may be more important in predicting help seeking.

Looking forward, this study sets milestones for the development and implementation of a short and concise profiling module in an existing or future mobile app for hearing screening linked to targeted recommendations, complementing the audiological assessment in a modular environment. In the context of this mobile study, individuals could provide any type of feedback in an open-question format at the end of the study. Of 185 individuals who completed the study, only 1 (0.005%) participant raised concerns related to usability and the user interface. This participant suggested enhancing the contrast between the fonts and the background and increasing the size of the click buttons. As this feedback was provided toward the end of our data collection period, we were unable to implement this suggestion in our design. Further studies that focus on user interface and usability are necessary for the future implementation of such a module in mHealth solutions that target an older population. Specific implementation strategies should be considered, such as simplicity of design; naturalness of navigation and task flow; clear interface elements; feedback [95,96]; large font sizes; contrasting colors; and clear, consistent, and simple instructions [11]. Perceived ease of use and perceived usefulness should be targeted to promote acceptance and use of mHealth solutions [11].

### Conclusions

This research provides initial knowledge regarding a selection of tests and questionnaires that have been shown to predict hearing help seeking in persons with self-reported hearing difficulties. From these, we derived conclusions for the implementation of an individual-profiling algorithm in an mHealth hearing app. This study is innovative in that it considers a comprehensive range of personal characteristics and covariates previously cited in the literature, including 25 assessment tools and 83 features, and narrows them down to identify a short selection of the most important predictors for profiling. Complementing the audiological assessments with such a profiling algorithm will enable an mHealth app to deliver targeted and efficient treatment recommendations depending on relevant individual characteristics. The benefits of such a profiling module might also extend to other functions within an mHealth hearing app. Future studies might explore potential relationships between psychological traits and, among others, HA fitting preferences and endurance in the fine-tuning process toward an optimal aiding solution; openness to try new, elaborate technical solutions; or preference for particular app usability features. We have seen how predictive models that use machine learning algorithms can be used to explore complex association patterns of individual characteristics and behaviors considering multiple predictors simultaneously and drawing robust conclusions through CV approaches. This provides further evidence of the advancement that the use of machine learning algorithms can bring to mHealth technology development [33]. mHealth solutions contribute to the evolution of hearing health care toward predictive, preventive, personalized, and participatory medicine (P4 medicine) [90]. We have seen how individual profiling in an mHealth hearing app can identify nonseekers, acting as a preventive action to reduce the risk of a late intervention for HL. It can also provide clinicians with data-driven insights on the individual health profile of the user for tailored and personalized treatments. Moreover, it can enhance the empowerment and participation of the individual in their own hearing health care, promoting informed decision-making. Indeed, personalization strategies (such as tailored treatment recommendations) increase the effectiveness of mHealth interventions [97]. To conclude, an mHealth hearing app that provides targeted treatment recommendations could facilitate faster access to hearing care services and subsequent earlier intervention where needed to pursue the long-term goal of achieving “hearing for all.”

## Appendix

Multimedia Appendix 1 Study design overview.

Multimedia Appendix 2 Hearing test feedback.

Multimedia Appendix 3 Feature importance values.

Multimedia Appendix 4 Feature correlation plots.

## Abbreviations

API: application programming interface
CV: cross-validation
HA: hearing aid
HL: hearing loss
MCC: Matthews correlation coefficient
mHealth: mobile health
NA: not available
NB: naïve Bayes
RF: random forest
RQ: research question
SNR: signal-to-noise ratio
SRT: speech recognition threshold
SVM: support vector machine

## Data Availability

The data sets generated during and analyzed during this study are available from the corresponding authors on reasonable request. Analysis scripts are available in the Zenodo repository [98]. A preprint of the manuscript was published on medRxiv [99] in February 2023 and revised in August 2023. Preliminary results of this research have been presented at symposia and conferences of the Hearing4All Cluster of Excellence, at the Virtual Conference of Computational Audiology 2022, and at the German Society for Psychology Congress 2022. The data will be shared with interested researchers upon request as the data set will be used for further projects and cannot be fully shared at the current time point.
