# Supplementary material for: Prediction of Hearing Help Seeking to Design a Recommendation Module of an mHealth Hearing App: Intensive Longitudinal Study of Feature Importance Assessment
Source: JMIR Hum Factors. 2024 Aug 12;11:e52310. doi: 10.2196/52310 (PMC11347899; doi:10.2196/52310)
Supplement: Multimedia Appendix 1 [file humanfactors_v11i1e52310_app1.pdf]

Multimedia appendix 1 – Study design

Baseline assessment (week 1)

	Questionnaire / Task
**Day 1**	Socio-demographic questionnaire
	Speech, Spatial and Qualities of hearing questionnaire (SSQ)
	Geriatric Anxiety Inventory (GAI)
	Digital literacy scale (Technikbereitschaft - Kurzskala)
	High Sensitive Person Scale (HSPS-G)
**Day 2** (7 am)	Vocabulary task
	Hearing Handicap Inventory for the Elderly and Adults (HHIE/A)
	Health Locus of Control questionnaire (Kontrollüberzeugungen zu Krankheit und Gesundheit – KKG)
	European Health Literacy questionnaire, short form (HLS-EU-Q16)
	Geriatric Depression Scale (GDS)
**Day 3** (7 am)	Berlin Fluid and Crystallized Intelligence task (BEFKI)
	Social Network index
	Expected Consequences of Hearing-Aid Ownership questionnaire (ECHO)
	De Jong Gierveld Loneliness scale
	Locus of Control scale (Internale-Externale Kontrollüberzeugung-4 Skala)
**Day 4** (7 am)	Attitudes towards Loss of Hearing Questionnaire (ALHQ v. 3.0)
	Big 5 personality traits (NEO Five-Factor Inventory)
	General health questionnaire (Fragebogen zum Allgemein Gesundheitzustand SF-12)
	Generalized Self-Efficacy Scale (GSES)
	Weinstein Noise Sensitivity Scale (WNSS)
**Day 5** (7 am)	Attitude to Ageing Questionnaire (AAQ)
	Perceived Stress Scale (PSS)
	Optimismus / Pessimismus scale (Die Skala Optimismus-Pessimismus -2)
	Sound Preference & Hearing Habits Questionnaire (SP-HHQ)

Longitudinal assessment (week 2 and 3)

	Questionnaire / Task
**Morning** (7 am)	Questions on sleep quality
	Daily questionnaire on affect
	Digit Triplet Test (DTT)
	Daily questionnaire on affect
**Evening** (7 pm)	Questions on special daily events
	Daily questionnaire on affect
	Digit Triplet Test (DTT)
	Daily questionnaire on affect

Multimedia appendix 1.2 – Longitudinal assessment details

Affect questionnaire

The affect questionnaire included 14 items in line with the Circumplex Model of Affect (Russel, 1980). Eight items were related to negative affect and six to positive affect. The items were displayed in a randomized order at each presentation and respondents had to indicate on a seven-point scale how much the specified mood applied to them (1 = does not apply; 7 = applies fully). For each of the English affective states retrieved from the Circumplex Model of Affect (Russel, 1980), two German synonyms were selected with the help of native German speakers.

	English item (Circumplex Model)	German item-pair implemented	
Negative affect items	nervous	angespannt	nervös
	sad	traurig	bekümmert
	upset	ärgerlich	entrüstet
	stressed (item not present in the model)	gestresst	gereizt
Positive affect items	excited	begeistert	hocherfreut
	happy	fröhlich	glücklich
	calm	entspannt	gelassen

Hearing test

Digit Triplets Test (DTT) – provided by the Hörzentrum Oldenburg gGmbH and publicly available under https://www.hz-ol.de/en/ztt.html

Further details on the test:
The speech material of the DTT is represented by random combinations of three digits (eg. "two six five") spoken by a female voice; all numbers between 0 and 9 are included, except from the disyllabic digit 7. The noise of the DTT consists of 30-time superimposition of speech material. Each hearing-test measurement consists of several presentations of spoken digits in noise, that the participant is asked to recognise and repeat on a number pad, for a total testing time of approximately three minutes. The test starts at a +4 dB SNR, so that the speech can be easily recognized by most participants. The SNR is then adjusted at every trial with a one-up one-down adaptive procedure with 2 dB step-size until the individual's SRT can be determined: after each trial, the noise increases if the participant's response is correct, while decreases if it is not. If a maximum value of +10 dB SNR is reached, the test is aborted and the result is categorized as NA. The measurement test error is of 0.7 dB. Participants were invited to familiarize with the test before starting the longitudinal assessment. They were instructed to perform the test with the use of their personal headphones, but three participants reported technical difficulties with their headphones and used loudspeakers instead.

Performance categorization and feedback	SRT < -7.1 d B SNR	Good performance (green feedback)
	-7.1 >= SRT < -5.1 dB SNR	Intermediate performance (yellow feedback)
	SRT >= -5.1 dB SNR	Poor performance (red feedback)
