# Supplementary material for: Prediction of Hearing Help Seeking to Design a Recommendation Module of an mHealth Hearing App: Intensive Longitudinal Study of Feature Importance Assessment
Source: JMIR Hum Factors. 2024 Aug 12;11:e52310. doi: 10.2196/52310 (PMC11347899; doi:10.2196/52310)
Supplement: Multimedia Appendix 2 [file humanfactors_v11i1e52310_app2.pdf]

# Multimedia appendix 2 – Hearing test feedback

Fk. VI | Department für Psychologie

Psychologische Methodenlehre und Statistik

Navigation: [...] > Psychologie ⊞ > Abteilungen ⊞ > Psychologische Methodenlehre und Statistik ⊞ > Testergebnis1

DEPARTMENT OF PSYCHOLOGY
CARL VON OSSIETZKY UNIVERSITY OLDENBURG


Kontakt

Leitung

Prof. Dr. Andrea Hildebrandt
+49 (0)441 798-4629
andrea.hildebrandt@uol.de
A07 0-062

Sekretariat

Sandra Marienberg
+49 (0)441 798 -5523
sandra.marienberg@uol.de
A07 0-035

Anschrift

Carl von Ossietzky Universität Oldenburg
Fakultät VI - Medizin und Gesundheitswissenschaften
Abt. Psychologische Methodenlehre und Statistik
Dep. für Psychologie


## Hörtestergebnis

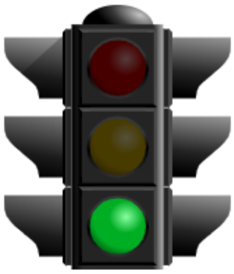

Ihr Hörvermögen bei diesem Hörtest ist normal. Dieser Hörtest kann natürlich nicht alle Aspekte Ihres Hörvermögens abdecken und ersetzt keine medizinische Diagnose. Wenn Sie trotzdem das Gefühl haben, schlecht zu hören, dann sollten Sie einen Hals-Nasen-Ohren-Arzt oder Hörgeräteakustiker konsultieren.

Das Verfahren ersetzt keine medizinische Diagnose, prüft aber das Hörvermögen in einer alltäglichen Situation und kann daher Hinweise geben, wie es um Ihr Gehör bestellt ist.

## Hörtestergebnis

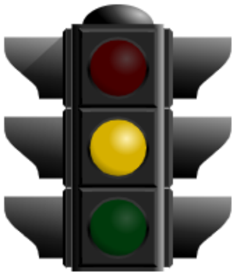

Bei diesem Hörtest verstehen die meisten Menschen etwas besser als Sie. Dieser Hörtest kann natürlich nicht alle Aspekte Ihres Hörvermögens abdecken und ersetzt keine medizinische Diagnose. Falls Sie eine weitergehende Beurteilung Ihres Hörvermögens wünschen, können Sie dafür einen Hals-Nasen-Ohren-Arzt oder Hörgeräteakustiker konsultieren.

Das Verfahren ersetzt keine medizinische Diagnose, prüft aber das Hörvermögen in einer alltäglichen Situation und kann daher Hinweise geben, wie es um Ihr Gehör bestellt ist.

## Hörtestergebnis

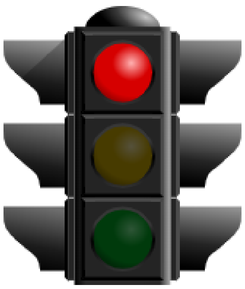

Bei diesem Hörtest verstehen die meisten Menschen deutlich besser als Sie. Dieser Hörtest kann natürlich nicht alle Aspekte Ihres Hörvermögens abdecken und ersetzt keine medizinische Diagnose. Für eine weitergehende Beurteilung Ihres Hörvermögens können Sie einen Hals-Nasen-Ohren-Arzt oder Hörgeräteakustiker konsultieren.

Das Verfahren ersetzt keine medizinische Diagnose, prüft aber das Hörvermögen in einer alltäglichen Situation und kann daher Hinweise geben, wie es um Ihr Gehör bestellt ist.
