# Supplementary material for: Prediction of Hearing Help Seeking to Design a Recommendation Module of an mHealth Hearing App: Intensive Longitudinal Study of Feature Importance Assessment
Source: JMIR Hum Factors. 2024 Aug 12;11:e52310. doi: 10.2196/52310 (PMC11347899; doi:10.2196/52310)
Supplement: Multimedia Appendix 3 [file humanfactors_v11i1e52310_app3.pdf]

Multimedia appendix 3 - Feature importance values

Feature set (global ranking)	Feature	RF importance value	RF rank	NB importance value	NB rank
Top-10	motivation to seek help (pre-study)	3.543	3	100.00	1
Top-10	HA expectations (global score)	3.813	1	86.614	3
Top-10	HA stigma (denial of HL scale)	2.891	5	90.945	2
Top-10	hearing performance M	2.907	4	74.349	4
Top-10	general attitude towards HA (pre-study)	2.461	6	72.168	5
Top-10	hearing feedback type: poor	1.639	9	71.835	6
Top-10	emotional consequencies HL	1.69	8	65.415	8
Top-10	self-reported hearing difficulties (qualities of hearing)	1.728	7	60.448	10
Top-10	source of motivation to seek help (pre-study)	1.116	21	62.659	9
Top-10	sensory processing sensitivity (ease of excitation scale)	1.549	10	16.596	47
Top-10	physical health	1.399	14	68.231	7
Top-15	neuroticism	1.542	11	38.916	23
Top-15	monthly income	0.667	57	57.208	11
Top-10	hearing performance SD	3.74	2	42.126	20
Top-15	social consequencies HL	0.732	54	51.878	13
Top-15	negative affect post-test M	1.436	13	13.749	55
Top-15	self-reported hearing difficulties (speech hearing)	1.338	15	52.635	12
Top-15	high sensitivity: sensory threshold	1.489	12	48.516	15
Top-15	HA stigma (negative coping strategies)	0.791	51	51.757	14
Top-20	mental health	1.08	26	46.457	16
Top-20	conscientiousness	1.335	16	11.296	58
Top-20	negative affect post-test SD	1.281	17	20.291	45
Top-20	hearing habits (unpredictable sounds)	0.869	41	44.034	17
Top-20	self-reported hearing difficulties (spatial hearing)	1.114	22	43.489	18
Top-20	general self-efficacy (global score)	1.243	18	9.7214	63
Top-20	social life participation (number of people)	1.14	19	10.842	60
Top-20	HA stigma (hearing-related esteem)	0.408	71	43.095	19
Top-20	noise sensitivity (global score)	1.125	20	14.991	50

Note: *HL: hearing loss; HA: hearing aids; M: mean; SD: standard deviation; NB: Naïve Bayes; RF: Random Forest*
