# Supplementary material for: Prediction of Hearing Help Seeking to Design a Recommendation Module of an mHealth Hearing App: Intensive Longitudinal Study of Feature Importance Assessment
Source: JMIR Hum Factors. 2024 Aug 12;11:e52310. doi: 10.2196/52310 (PMC11347899; doi:10.2196/52310)

Multimedia appendix 4 – Correlation plots

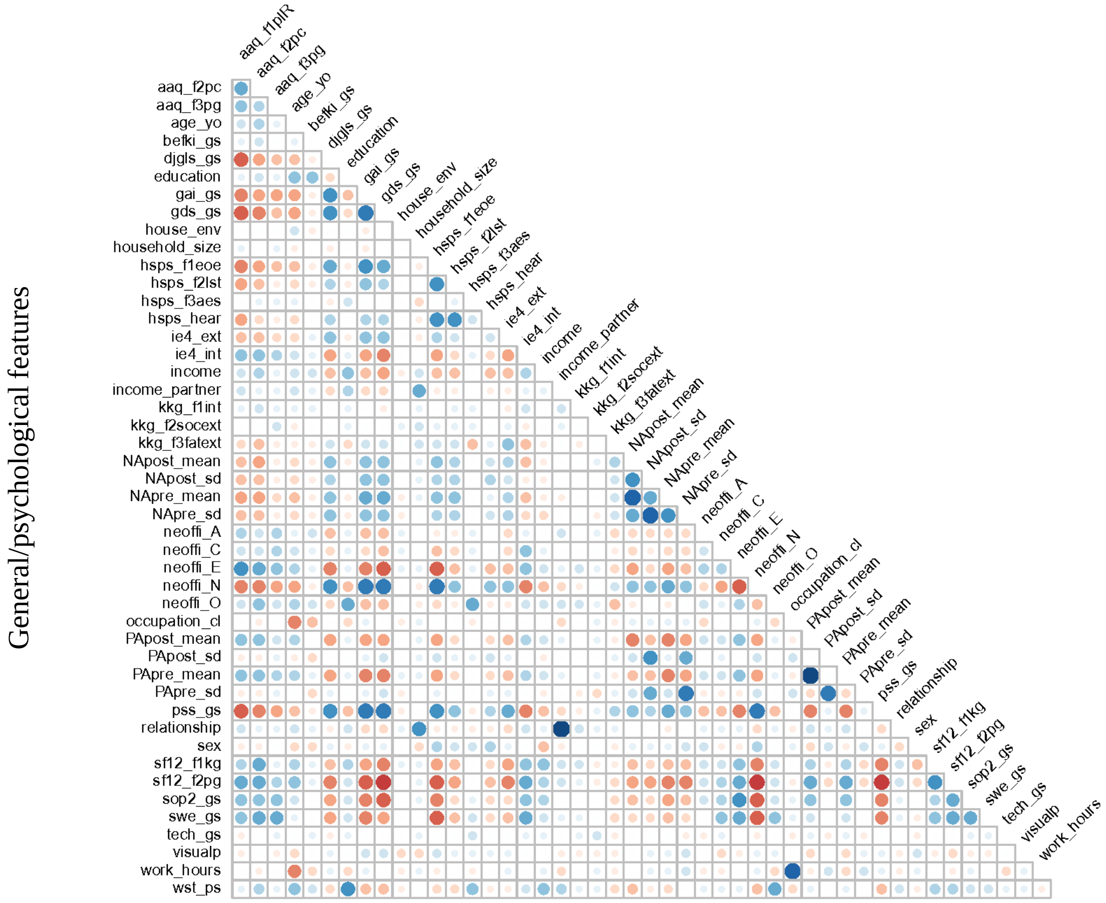

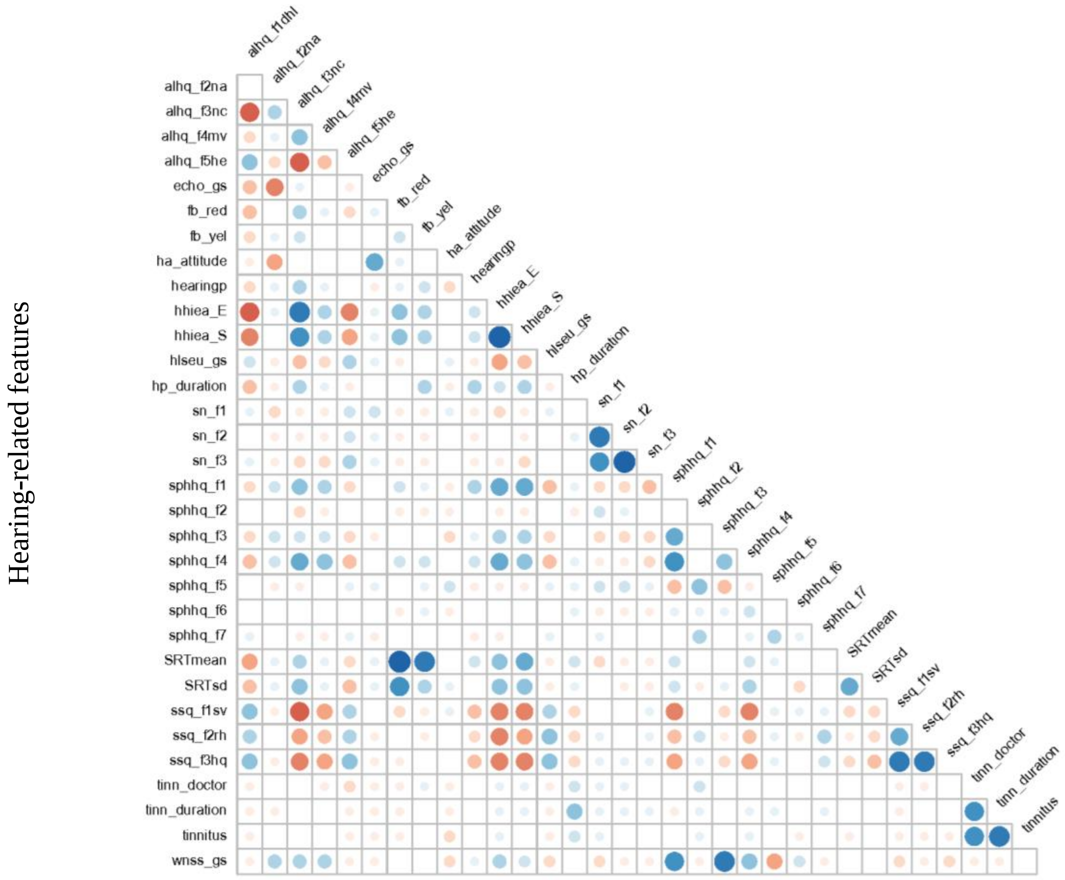

Supplement: Multimedia Appendix 4 [file humanfactors_v11i1e52310_app4.pdf]
